# A Text-Based Smoking Cessation Intervention for Sexual and Gender Minority Groups: Protocol for a Feasibility Trial

**DOI:** 10.2196/42553

**Published:** 2022-12-09

**Authors:** Irene Tami-Maury, Rebecca Klaff, Allison Hussin, Nathan Grant Smith, Shine Chang, Lorna McNeill, Lorraine R Reitzel, Sanjay Shete, Lorien C Abroms

**Affiliations:** 1 School of Public Health The University of Texas Health Science Center at Houston Houston, TX United States; 2 Department of Psychological, Health, and Learning Sciences University of Houston Houston, TX United States; 3 MD Anderson Cancer Center The University of Texas Houston, TX United States; 4 Milken Institute of Public Health The George Washington University Washington, DC United States

**Keywords:** smoking cessation, sexual and gender minorities, LGBTQ+, SMS text messaging, mobile health, mHealth

## Abstract

**Background:**

Smoking among sexual and gender minority (SGM) groups, which include lesbian, gay, bisexual, transgender, and queer individuals, has been reported to be highly prevalent. This is attributed to several factors, including minority-specific stress and targeted tobacco marketing. Therefore, this population is at an increased risk for tobacco-related diseases. SMS text messaging programs have been found to be effective for smoking cessation and appeal to traditionally hard-to-reach populations over other interventions. It has also been suggested that targeted and tailored interventions could be more effective among SGM smokers because they can be designed to assure a safe, validating health care environment that enhances receptivity to cessation.

**Objective:**

The aim of this study is to develop SmokefreeSGM, a text-based smoking cessation program tailored to and tested among SGM smokers.

**Methods:**

The study consists of three phases, culminating in a feasibility trial. In Phase 1, our research team will collaborate with a Community Advisory Board to develop and pretest the design of SmokefreeSGM. In Phase 2, the tailored text messaging program will be beta tested among 16 SGM smokers. Our research team will use a mixed-methods approach to collect and analyze data from participants who will inform the refinement of SmokefreeSGM. In Phase 3, a feasibility trial will be conducted among 80 SGM smokers either enrolled in SmokefreeSGM or SmokefreeTXT, the original text-based program developed by the National Cancer Institute for the general population. Our research team will examine recruitment, retention, and smoking abstinence rates at 1-, 3-, and 6-month follow-up. Additionally, a qualitative interview will be conducted among 32 participants to evaluate the feasibility and acceptability of the programs (SmokefreeSGM and SmokefreeTXT).

**Results:**

This study received approval from The University of Texas Health Science Center at Houston Committee for the Protection of Human Subjects to begin research on August 21, 2020. Recruitment for the beta testing of SmokefreeSGM (Phase 2) began in January 2022. We estimate that the feasibility trial (Phase 3) will begin in September 2022 and that results will be available in December 2023.

**Conclusions:**

Findings from this research effort will help reduce tobacco-related health disparities among SGM smokers by determining the feasibility and acceptability of SmokefreeSGM, an SGM-tailored smoking cessation intervention.

**Trial Registration:**

ClinicalTrials.gov NCT05029362; https://clinicaltrials.gov/ct2/show/NCT05029362

**International Registered Report Identifier (IRRID):**

DERR1-10.2196/42553

## Introduction

### Background

Cigarette smoking among sexual and gender minority (SGM) groups in the United States is higher than heterosexual and cisgender individuals. Nearly 1 in 4 SGM adults smoke cigarettes compared with about 1 in 6 heterosexual adults [1]. The National Institutes of Health define SGM as an umbrella term that includes individuals who identify as lesbian, gay, bisexual, asexual, transgender, Two-Spirit, queer, or intersex. It also includes individuals with same-sex or same-gender attractions or sexual behaviors and those with a difference in sex development, as well as those whose sexual orientation, gender identity or expression, or sex development is characterized by nonbinary constructs [2]. The high rate of cigarette smoking among SGM groups is attributed to several factors, including additional stress due to stigmatization and discrimination, as well as targeted tobacco marketing [3]. Therefore, this population is at an increased risk for developing tobacco-related health conditions, including heart disease and stroke. Because smoking accounts for at least 30% of all cancer deaths, SGM individuals are also at an increased risk of suffering from this fatal disease as a result of their smoking behaviors [4].

It has been suggested that targeted and tailored interventions could be more effective among SGM smokers because they assure a safe, validating environment that enhances receptivity to cessation [5,6]. The few reported smoking cessation interventions for SGM smokers are minimally tailored, lack a control group, lack objective verification of self-reported quit rates, or are based on group interventions [7-10]. Mobile health (mHealth) programs that use SMS text messaging have been found effective for smoking cessation and other behavior change interventions [11-13]. These programs are appealing to marginalized groups who experience barriers to smoking cessation interventions and who have high rates of mobile phone and text messaging use because they allow for self-management and discretion [11-14]. Although the number of people enrolled in text messaging programs for smoking cessation is increasing, no study has evaluated their feasibility, specifically among SGM smokers.

SmokefreeTXT is an automated, personalized, and interactive mHealth program for smoking cessation developed by the National Cancer Institute (NCI), which sends supportive text messages 2 weeks before and up to 6 weeks after a quit date. SmokefreeTXT has been successfully tested among the general population [15]. It has also been successfully tailored to special populations such as pregnant women, homeless individuals, and veterans [11,16-19]. However, theoretically-based smoking cessation treatments delivered via text messaging and focused on enhancing treatment engagement and targeting the specific needs of SGM smokers are needed.

This project is guided by a conceptual framework consistent with existing models and health outcomes, models of SGM health disparities, and social cognitive models of smoking cessation [20-24]. We hypothesize that sociodemographic features, particularly sexual orientation and gender identity, will determine level of stress, systematic harassment and discrimination that will thereby influence individuals’ access to resources, experience of stressful events, and predisposed vulnerabilities. These factors will serve as immediate precipitants of smoking episodes.

The proposed project provides an advantageous opportunity to test the feasibility of a promising cessation practice for SGM smokers supported by mobile phone text messaging. SGM individuals have been identified as groups at elevated risk for cancer, in part because of their high smoking rates, which are largely reflected by the tobacco industry’s long history of targeting SGM communities [4]. Furthermore, factors such as low rates of health insurance coverage, high rates of stress due to systematic harassment and discrimination, and a low level of SGM-related cultural competency in the health care system have negatively affected the health of SGM individuals, as well as their access to smoking cessation treatments, including counseling and medication [25].

The rapid increase in the number of people, including SGM individuals owning a mobile phone, has led to the development of new apps in the self-management of chronic diseases and behavior change interventions [26]. The proposed text-based smoking cessation intervention will include motivational messages or content specifically tailored to SGM participant characteristics to distract from cravings and will be sent based on participants’ needs. While there is strong evidence that text-based tobacco cessation interventions help smokers quit smoking, no similar intervention has been conducted specifically among SGM smokers [27-29]. Reducing smoking prevalence among SGM individuals by implementing a cost-effective and tailored text-based smoking cessation intervention is a powerful cancer prevention strategy for this population.

### Objective

The objective of the study is to develop SmokefreeSGM, an SGM-tailored version of SmokefreeTXT that will be tested among SGM smokers. This protocol proposes 3 aims, each corresponding to Phases 1, 2, and 3 of this study:

- Aim 1: to develop an SGM-tailored text-based smoking cessation program and assess the readability and acceptability of its text messages
- Aim 2: to beta test the design of SmokefreeSGM through a mixed-methods approach among 16 SGM adult smokers
- Aim 3: to conduct a two-arm (SmokefreeSGM vs SmokefreeTXT) randomized controlled trial for examining recruitment, retention, and smoking abstinence rates at 1-, 3-, and 6-month follow-up among 80 SGM smokers

A qualitative interview will be conducted for 32 participants to evaluate the feasibility (recruitment and retention rates) and acceptability of the programs.

## Methods

### Study Design

#### Phase 1

SmokefreeTXT is a text-based smoking cessation program developed for the general population by the NCI. SmokefreeTXT is a personalized and interactive mHealth program that sends bidirectional text messages timed around a participant’s quit date over 2 months. The text messages include pre- and postquit educational messages, peer ex-smoker messages, nicotine replacement therapy medication reminders, and relapse messages. Messages are based on social cognitive theory and are consistent with the US Public Health Service Clinical Practice Guideline [30,31]. Messages are interactive and prompt users to track smoking, report cravings, and provide their smoking status. Participants who report that they have not quit are routed into setting a new quit date. SmokefreeTXT offers both outgoing messages and on-demand help through the use of keywords, including CRAVE (user will receive help with cravings by having a reminder of why they should not smoke), MOOD (user will receive a positive message when having a difficult day), SLIP (user will receive extra encouragement to get back on track), and SMOKEFREE STATUS (indicates if the user has smoked or not by the time they receive the text message).

To develop the SmokefreeSGM, our research team will collaborate with self-identified SGM individuals, tobacco specialists, and scientists and clinicians engaged in SGM research. This will allow us to create encouraging and motivational messages that address unique psychosocial stressors for SGM smokers such as elevated general stress and minority-specific stress (ie, internalized homophobia, sexual orientation concealment, and discrimination events). A new keyword, STRESS, will be created to prompt these additional set of text messages that will be sent from a fictitious peer SGM ex-smoker named Alex who offers evidence-based advice on quitting. The messages sent by Alex will be based on real-life experiences of SGM ex-smokers who understand the user’s barriers in order to create a welcoming environment.

#### Phase 2

Once the SmokefreeSGM program is developed, it will be beta tested among 16 SGM smokers through a mixed-methods approach.

#### Phase 3

A feasibility trial will be conducted to examine recruitment, retention, and smoking abstinence rates at 1-, 3-, and 6-month follow-up among 80 SGM smokers randomized to either the SmokefreeTXT or SmokefreeSGM program.

### Study Population and Recruitment Strategy

#### Phase 1

Not applicable, as no human subjects will be involved.

#### Phase 2

The SmokefreeSGM text messaging program will be beta tested among 16 self-identified SGM individuals currently living in Texas. Various efforts on behalf of our research team will be made to identify individuals suitable for enrollment in the study. Flyers offering help to SGM individuals interested in quitting smoking will be distributed at local community organizations and health care facilities working with and for this population in Texas. Information about this part of the study will also be posted in local newspapers and magazines, as well as on web pages and social media sites of local community organizations, health care facilities, and SGM venues (eg, bars and restaurants).

#### Phase 3

A total of 80 SGM individuals currently living in Texas will be recruited to participate in the feasibility trial through similar efforts to those used by our research team in the beta test (Phase 2). Participants will be randomized into the SmokefreeTXT (n=40, 50%) or SmokefreeSGM (n=40, 50%) text messaging program. Efforts will be made to proportionally balance the study sample with each one of the major SGM subgroups—lesbian, gay, bisexual, and transgender individuals.

### Eligibility Criteria

#### Phase 1

Not applicable.

#### Phases 2 and 3

The inclusion criteria for participants in the beta test (Phase 2) and feasibility trial (Phase 3) are as follows: (1) self-identifies as an SGM individual, (2) is aged ≥18 years, (3) smokes 5 or more cigarettes per day (smoking status biologically confirmed by saliva cotinine test), (4) has an interest in quitting smoking in the next 15 days, (5) has a cellphone number with an unlimited short messaging service plan, (6) has US mailing and email addresses, and (7) provides positive cotinine saliva test results.

The exclusion criteria for participants in study Phases 2 and 3 are as follows: (1) has a prepaid cell phone, (2) has a cellphone number that does not work or is registered to someone else, (3) is pregnant or breastfeeding, (4) has contraindication for nicotine patches, (5) has current use of tobacco cessation medications, (6) is enrolled in another smoking cessation study, (7) is a non-English speaker, and (8) has inadequate equipment or device (eg, webcam, speakers, and mic) for participating in telehealth sessions. It is important to note that those individuals participating in Phase 2 of the study will be excluded from Phase 3 to reduce the risk of bias.

### Data Collection

#### Phase 1

After completion of the text library for SmokefreeSGM, the Flesch-Kincaid Grade Level and the Dale-Chall scores will be calculated using a web-based tool (datayze.com) to determine the readability of each text message. The Flesch-Kincaid Grade Level assesses the approximate reading grade level of each text message based on sentence length (average number of words in a sentence) and word length (average number of syllables in a word) [32]. The Dale-Chall score assesses the readability of text based on a list of 3000 words commonly understood by 4th graders [33]. Both measures will help us determine whether the average adult would be able to comprehend the content of the SmokefreeSGM text messages and allow us to make changes where necessary.

The text library will then be input into a web-based survey to collect suggestions and feedback from our advisory board members (eg, SGM smokers, tobacco specialists, and SGM researchers and clinicians). Experts will rate each text message on a scale of 1 to 5, where 1 indicates *Totally Unacceptable* and 5 indicates *Perfectly Acceptable*. The mean, median, and range of these values will be calculated. Experts will also be able to provide additional feedback in a designated comment box. Data collected from these surveys will help us determine what revisions are needed. The revised text library will be input into a text messaging platform and internally tested by research staff.

#### Phase 2

Immediately following confirmation of the participant’s eligibility and enrollment into the study, a baseline assessment will be completed, and an 8- or 10-week supply of nicotine patches (according to each study participant’ smoking status) will be sent to the participant via mail. The baseline web-based questionnaire will include items assessing demographic (eg, age, gender, sexual orientation and gender identity, race or ethnicity, and education) and smoking characteristics of the participants (eg, lives with one or more smokers in the household, cigarettes smoked per day, and past quit attempts). Nicotine dependence will be measured with the Fagerstrom Test for Nicotine Dependence [34].

A follow-up survey at 1 month after the participant’s quit date (6 weeks following screening) will include measures of smoking cessation through self-report of 7-day smoking abstinence [35]. A 10-item questionnaire, the System Usability Scale (SUS), will be used to assess the usability of SmokefreeSGM. This will be complemented by a structured interview where qualitative data on the usability will be collected among study participants. For this purpose, the SUS will be used because it is a quick and cost-effective yet accurate approach to assessing usability [36].

#### Phase 3

Individuals interested in the study will be screened and if eligible, consented. Those individuals enrolled into the study will be randomized into either the SmokefreeTXT (n=40) or SmokefreeSGM (n=40) text messaging program. All participants will receive their 8- or 10-week supply of nicotine patches via mail. The baseline survey will include the same items included in Part 2. Recruitment will be assessed as explained above. Retention and smoking abstinence rates will be examined at 1-, 3-, and 6-month follow-up. Smoking abstinence outcome will be defined as 7-day smoking abstinence along with a negative saliva cotinine test.

Additionally, structured web-based interviews will be conducted with a subsample of 32 study participants completing the feasibility trial. Participant satisfaction with the program will be assessed by a series of questions in which participants will be asked to comment on the text messages (eg, “Could you tell us if the program was helpful or not in getting you to try to quit?” “What ideas on how to quit did you like the most about the program?” and “Would you recommend the program to a friend interested in quitting? Why?”). Participants will be also asked to make suggestions for improving the program and note which features they liked and disliked. Questions will be open-ended to elicit qualitative feedback. Some open-ended probes will be used to learn why a participant responded a certain way to the keywords; for example: “When and why did you text CRAVE?” “How would you improve some of the text messages?” “How do you feel about your ability to remain smoke-free?” and “Can you tell me if there was anything confusing about the texts?” Concepts will be discussed with the study participants until data reach saturation.

### Analysis

#### Phase 1

The Flesch-Kincaid Grade Level test uses a formula that depends on sentence length and word length. The formula is as follows: 0.39 x (words/sentences) + 11.8 x (syllables/words) – 15.59. The resulting score corresponds with the grade level needed to understand the content. A resulting score ≥80 means that the text message is easy or very easy to read [32]. The Dale-Chall score is another readability test that uses a list of 3000 words that groups of 4th grade American students could reliably understand. A score of <7.0 means that the text is easily understood by an average 8th grade student or lower [33]. Our research team will verify that each text message created for the SmokefreeSGM program has a Flesch-Kincaid Grade Level score of ≥80 and a Dale-Chall score of <7.0 to ensure that they will be easily understood by all users.

#### Phase 2

The recruitment rate will be defined by dividing the number of SGM smokers who consent and complete the baseline assessment by the number of SGM smokers who are approached and invited to participate in the text-based program. Similarly, the retention rate will be defined by dividing the number of SGM smokers who remained in the text-based cessation program and completed the 1-month assessment by the number of SGM smokers recruited. Additionally, 1 month following the quit date, participants will take part in a structured interview to collect both quantitative and qualitative data using the SUS. After each participant assigns points—Strongly Disagree (1) to Strongly Agree (5)—to each of the 10 items of the scale, a score is calculated. SUS is scored on a 0-100 scale. A score of 74 or more will indicate that SmokefreeSGM has high perceived usability [36]. It is important to note that after the participants score each SUS item, the moderator will ask “Why did you assign this many points to this question?” to obtain the corresponding qualitative data. The audio recordings from these responses will be transcribed for qualitative analysis. Qualitative data will be analyzed using a descriptive framework approach. which allows for the exploration of prior concepts and for new themes to emerge [37]. Transcripts will be read and reread to gain familiarity with the subject. Analysis of the transcripts will be based on data grouping, creation of a code guide, and identification of themes from the narrative text. The code guide will be drawn from the domains shaped by the discussion guide and themes that will emerge during the study. The method of constant comparison (comparing concepts across categories to identify links, patterns, connections, and differences) will be used to identify themes within the data. Themes will be compared with theoretical constructs in the conceptual framework to determine if the model captures the phenomena of interest and to inform the refining of the SmokefreeSGM design.

#### Phase 3

The recruitment rate will be calculated as explained above. Retention rate will be defined by dividing the number of SGM smokers who remained in the text-based cessation program and completed the 3- and 6-month assessments by the number of SGM smokers recruited. The recruitment and retention rates will be calculated along with CIs for both arms (both arm-specific and combined arms). Targeted recruitment and retention rates are not considered in this protocol paper as they vary considerably in smoking cessation interventions (4% to 95% and retention rates from 36% to 100%) [38]. However, we will use *t* test or Mann-Whitney, on the one hand, or chi-square or Fisher exact test, on the other, to compare baseline demographic variables between SGM smokers and those who refuse to participate or drop out from the study. Additionally, we will assess the predictors of recruitment and attrition rates across study participants.

Smoking abstinence outcome will be defined as 7-day smoking abstinence along with negative saliva cotinine. Because abstinence is a binary variable (yes or no) the primary method of analysis will be a simple posttest analysis among SGM smokers with a generalized logistic mixed model, using a random intercept to incorporate the intrasubject correlation. Important covariates that will be used to adjust for potential baseline differences include age, level of education, biological sex, marital status, gender identity or sexual orientation, race or ethnicity, working status, health insurance, income, years of smoking, smoking initiation, nicotine dependence, use of other tobacco products, and living with other household members who smoke. Odds ratio of smoking abstinence in the SmokefreeSGM group relative to the SmokefreeTXT group will be used to estimate effect size. Using logistic regression, abstinence will be regressed onto a dummy coded SmokefreeTXT versus SmokefreeSGM group indicator, with the exponentiated coefficient of the group indicator yielding the odds ratio.

Once the smoking cessation intervention concludes and in order to quantitatively assess engagement, a series of 32 individual interviews will be conducted with individuals previously enrolled in the feasibility trial. In this sense, the number of text messages a participant sends to the computer system, including replies to the SmokefreeTXT and SmokefreeSGM programs and keywords used, will be totaled, and averages will be calculated across participants. The total will not include use of the keyword STOP, a keyword for unsubscribing from the program. The percentage of participants who use this keyword will serve as an indicator of program disengagement. We will examine means and standard deviations of the number of text messages over the course of the 6-month program in each arm. We will use *t* test or Mann-Whitney to compare the average numbers of text messages from the two arms.

Transcripts from the qualitative interviews will be analyzed following the procedures described for the structured interviews in Part 2.

Quantitative data will be analyzed using computer software (Excel [Microsoft Corporation], SPSS [IBM], R [R Foundation], SAS [SAS Institute], or STATA [StataCorp]). Qualitative data analysis will be performed using computer software (ATLAS.ti), which will significantly reduce the amount of time needed for coding and categorizing qualitative data.

### Ethics Approval

This study received Institutional Review Board approval (HSC-SPH-0318) from The University of Texas Health Science Center at Houston Committee for the Protection of Human Subjects.

This study involves no more than minimal risk to subjects.

## Results

This study has been designed to develop an SGM-tailored smoking cessation text program (SmokefreeSGM) and test its feasibility among SGM smokers. Study findings will contribute to reducing tobacco-related health disparities among SGM groups.

Principal investigator (IT-M) received approval from UTHealth Committee for the Protection of Human Subjects to begin research on August 21, 2020, and was awarded funding from the NCI on September 1, 2020. The development of the SmokefreeSGM program has been completed, and recruitment for beta testing began in January 2022. We estimate that recruitment for the feasibility trial will begin in September 2022 and that results will be available in December 2023.

## Discussion

### Overview

We anticipate that the findings of our feasibility trial will support the need for SGM-tailored smoking cessation interventions and mHealth tools. SmokefreeSGM is not only cost-effective and user friendly but allows for personalized care and self-management. Findings from our feasibility trial will be used to design and implement large-scale mHealth-based interventions to address the high prevalence of cigarette smoking and tobacco-related health disparities among SGM individuals. Additionally, our findings will help inform future text-based smoking cessation studies for SGM groups and other marginalized populations and contribute to the body of evidence for mHealth behavior change interventions.

### Generalizability and Limitations

While we have limited recruitment to SGM individuals living in Texas, the state’s diversity enhances the generalizability of our findings. Texas ranks sixth in the country for highest diversity index, a measure of the probability that 2 people chosen at random will be of different racial and ethnic groups [39]. Furthermore, a recent analysis found Texas to be the second most diverse state in the country when accounting for the following categories: socioeconomic diversity, cultural diversity, economic diversity, household diversity, religious diversity, and political diversity [40].

We recognize that excluding Spanish speakers limits the potential generalizability and reach of our text-based smoking cessation program, considering that Latinx people represent approximately 40% of the total population in Texas [41]. However, we do believe that developing and delivering an SGM-tailored intervention in 2 languages (English and Spanish) will be the logical next step in our research programs.

We implemented procedures to limit biases that arise from conducting a randomized controlled trial. While the investigators will be aware of the study arm (SmokefreeTXT or SmokefreeSGM) that participants are enrolled in, participants will be masked to that information in order to mitigate performance bias. We will also be randomizing participants to the intervention conditions to help limit the influence of confounders and mitigate selection bias. Our outcome measures will rely on saliva cotinine tests, which will deter social desirability bias from incorrect self-reported cessation status from participants who have not quit smoking. To account for attrition bias due to possible differences in the number of withdrawals between study arms, we will include all participants who were randomized into the study in our data analysis, as opposed to only those who completed the entire intervention.

Regarding our proposed timeline, it should be noted that our study was first delayed because of the COVID-19 pandemic. This required us to revise and resubmit our protocol to account for data collection being moved to a virtual environment. Our efforts were also interrupted by the winter storm that impacted Texas in February 2021.

## Abbreviations

mHealth: mobile health
NCI: National Cancer Institute
SGM: sexual and gender minority
SUS: system usability scale

## Data Availability

Data sharing is not applicable for this article as data collection has not been completed, and data sets have not yet been generated for analysis in the current study.
